# Antiangiogenic activity of aplidine, a new agent of marine origin

**DOI:** 10.1038/sj.bjc.6601864

**Published:** 2004-06-01

**Authors:** G Taraboletti, M Poli, R Dossi, L Manenti, P Borsotti, G T Faircloth, M Broggini, M D'Incalci, D Ribatti, R Giavazzi

**Affiliations:** 1 Department of Oncology, Mario Negri Institute, Bergamo 24125, Italy; 2 Department of Oncology, Mario Negri Institute, Milan 10157, Italy; 3 Pharma Mar USA Inc., Cambridge, MA 02139, USA; 4 Department of Human Anatomy and Histology, University of Bari, Bari 70124, Italy

**Keywords:** aplidine, angiogenesis inhibition, endothelial cells, didemnin

## Abstract

The antineoplastic compound aplidine, a new marine-derived depsipeptide, has shown preclinical activity *in vitro* on haematological and solid tumour cell lines. It is currently in early phase clinical trials. The exact mechanism of action of this anticancer agent still needs to be clarified. We have previously reported that aplidine blocks the secretion of the angiogenic factor vascular endothelial growth factor (VEGF) by the human leukaemia cells MOLT-4, suggesting a possible effect on tumour angiogenesis. This study was designed to investigate the antiangiogenic effect of aplidine. *In vivo*, in the chick embryo allantoic membrane (CAM) assay, aplidine inhibited spontaneous angiogenesis, angiogenesis elicited by exogenous VEGF and FGF-2, and induced by VEGF overexpressing 1A9 ovarian carcinoma cells. *In vitro*, at concentrations achievable in the plasma of patients, aplidine inhibited endothelial cell functions related to angiogenesis. It affected VEGF- and FGF-2-induced endothelial cell proliferation, inhibited cell migration and invasiveness assessed in the Boyden chamber and blocked the production of matrix metalloproteinases (MMP-2 and MMP-9) by endothelial cells. Finally, aplidine prevented the formation of capillary-like structures by endothelial cells on Matrigel. These findings indicate that aplidine has antiangiogenic activity *in vivo* and inhibits endothelial cell functional responses to angiogenic stimuli *in vitro*. This effect might contribute to the antineoplastic activity of aplidine.

Aplidine, dehydrodidemnin B, is a marine-derived antitumour agent isolated from the Mediterranean tunicate *Aplidium albicans* (Sakai *et al*, 1996; da Rocha *et al*, 2001; Jimeno, 2002; Vera and Joullie, 2002). It is a cyclic depsipeptide, structurally related to other naturally occurring didemnins, such as didemnin B.

Aplidine was at first selected for its enhanced cytotoxicity against different tumour cells lines and lower myelotoxicity relative to didemnin B (Geldof *et al*, 1999; Albella *et al*, 2002). *In vitro*, aplidine inhibited tumour cell proliferation with an IC_50_ in the nanomolar/subnanomolar range, with complete inhibition achieved at 10 nM (Lobo *et al*, 1997; Erba *et al*, 2002; Erba *et al*, 2003). Short-term exposure to aplidine rapidly caused apoptosis of tumour cells (Erba *et al*, 2002; Gajate *et al*, 2003) and prevented the formation of colonies by explanted tumour cells (Depenbrock *et al*, 1998). In xenograft models, aplidine showed activity against different tumour types (Albella *et al*, 2002). Results from early phase clinical trials have confirmed the reduced myelotoxicity of aplidine relative to didemnin B, and have shown activity against certain tumour types such as medullary thyroid carcinoma, renal-cell carcinoma, melanoma and tumours of neuroendocrine origin (reviewed in Schwartsmann *et al*, 2001).

The molecular mechanism of action of aplidine still remains to be clarified, as different activities of the compound have been described that might potentially contribute to its antitumour activity (Sakai *et al*, 1996; Vera and Joullie, 2002). The antiproliferative effect of aplidine on cancer cells has been associated to perturbation in the cell cycle, since aplidine, at low concentrations, induces blockade of the cell cycle at G1 and at G2 (Geldof *et al*, 1999; Erba *et al*, 2002). Didemnins also block protein synthesis (Crews *et al*, 1994), inhibit ornithine decarboxylase, a regulator of intracellular polyamine levels (Urdiales *et al*, 1996; Gomez-Fabre *et al*, 1997; Erba *et al*, 2002) and bind to and inhibit the activity of the palmitoyil protein thioesterase, involved in signal transduction pathways associated to cell proliferation (Crews *et al*, 1996). However, the relevance of these activities in determining the antineoplastic activity of the compound is still debated.

We have previously reported that aplidine inhibited the secretion of vascular endothelial growth factor (VEGF) by the human leukaemia MOLT-4 cells, leading to blockage of the VEGF/VEGFR-1 autocrine loop that regulated MOLT-4 cell proliferation (Fusetti *et al*, 2000; Marchini *et al*, 2002; Broggini *et al*, 2003). Vascular endothelial growth factor is one of the major stimulators of angiogenesis, the formation of new blood vessels during physiological and pathological conditions (Folkman, 1995; Carmeliet and Jain, 2000). Angiogenesis is crucial in the progression of tumours, since the growth of the tumour mass and the process of metastasis depend on the existence of a functional blood supply system (Folkman, 1995; Carmeliet and Jain, 2000). Therefore, the inhibitory activity of aplidine on VEGF production suggested that the compound might exert an antiangiogenic effect by interfering with VEGF-mediated responses. This, in turn, might represent an additional mechanism of the antineoplastic activity of aplidine. This study was designed to further investigate the antiangiogenic activity of aplidine, in *in vitro* and *in vivo* models of angiogenesis.

## MATERIALS AND METHODS

### Aplidine

Aplidine (provided by PhamaMar, Madrid, Spain) was dissolved in DMSO or absolute ethanol (1000 × stock solution) and further diluted in the test medium immediately before the assay. In all the *in vitro* experiments, control cells received the same volume of DMSO (‘vehicle’). At the used dilution (at least 1 : 1000), DMSO had no effect on endothelial cell motility and proliferation. Similar results were obtained when aplidine was dissolved in DMSO or in absolute ethanol.

### Cells

Human umbilical vein endothelial cells (HUVECs) were isolated from umbilical cord veins and grown on 1% gelatin-coated flasks in M199 supplemented with 10% foetal calf serum (FCS), 10% newborn calf serum, 20 mmol l^−1^ HEPES, 6 U ml^−1^ heparin, 2 mmol l^−1^ glutamine, 50 *μ*g ml^−1^ endothelial cell growth factor (crude extract from bovine brain), penicillin and streptomycin. Cells were used between the third and fifth passage.

The human ovarian carcinoma 1A9 cells and its VEGF-overexpressing clone 1A9-VS4 were cultured in RPMI 10% FCS. 1A9-VS4, obtained by transfection of 1A9 cells with the human VEGF cDNA, was found to express four-fold higher levels of VEGF compared to parental 1A9 cells (manuscript in preparation). Tumour cells (10 × 10^6^) were injected subcutaneously in nude mice. Growing tumours were collected and fragments were grafted onto the chick embryo allantoic membrane (CAM).

Human proximal tubular epithelial cells HK-2, from the American Type Culture Collection (Rockville, MD, USA), were cultured as described (Morigi *et al*, 2002).

### Angiogenesis assay (chorioallantoic membrane assay)

Fertilised White Leghorn chicken eggs (10 per group) were incubated at 37°C at constant humidity. On day 3 of incubation, a square window was opened in the egg shell after the removal of 2–3 ml of albumen so as to detach the developing CAM from the shell. The window was sealed with a glass and the eggs were returned to the incubator. On day 8, 1 mm^3^ sterilised gelatin sponges (Gelfoam Upjohn, Kalamazoo, MI, USA) were placed on the top of the growing CAM, according to the method of Ribatti *et al* (1997). Then, the sponges were loaded with: (a) 1 *μ*l of phosphate-buffered saline (PBS) containing different concentrations of aplidine (1, 5, 10 and 20 nM); (b) 1 *μ*l of PBS or 1 *μ*l of PBS containing 500 *μ*g of recombinant human VEGF_165_ or FGF-2 (R & D Systems, Abingdon, UK) as negative and positive controls, respectively; and (c) 1 *μ*l of PBS containing 500 *μ*g of recombinant human VEGF_165_ or FGF-2 and 10 nM of aplidine.

In a second set of experiments, 1 mm^3^ bioptic fragments obtained from xenografts derived from 1A9 and 1A9-VS4 tumours (see above) were grafted onto the CAM on day 8. Tumour fragments were gently placed onto the CAM surface and care was taken to avoid injuring major blood vessels, as described (Knighton *et al*, 1977). At different times after grafting (4, 28, 52 and 72 h), embryos were treated with PBS or with 10 nM aplidine pipetted directly onto the implants.

CAMs were examined daily until day 12 and photographed *in ovo* with a stereomicroscope equipped with a Camera System MC 63 (Zeiss, Oberkochen, Germany). On day 12, blood vessels entering the sponges or the implants within the focal plane of the CAM were counted by two observers in a double-blind manner at × 50 magnification. The mean values±standard deviation (s.d.) for vessel counts were determined for each analysis.

### Endothelial cell proliferation assay

To assess proliferation (Belotti *et al*, 1996; Taraboletti *et al*, 2002), HUVECs (4 × 10^3^ cells well^−1^) were plated in a 96-well plate in a medium containing 5% serum. After 24 h, FGF-2 or VEGF (10 ng ml^−1^) were added, together with the compound. Vehicle or aplidine (0.02–20 nM) were incubated for 1, 24 or 72 h. After washing, cells were incubated in the medium supplemented with FGF-2 or VEGF for a total 3 days. Cells were fixed and stained with 0.5% crystal violet in 20% methanol, rinsed and air-dried. The stain was eluted with ethanol : 0.1 M sodium citrate solution (1 : 1) and absorbance at 540 nm was measured with a Multiscan MC Titertek (Flow Laboratories, Milan, Italy). Data are expressed as the percentage of control proliferation (vehicle-treated cells) and as the IC_50_ (drug concentration causing 50% inhibition).

### Endothelial cell motility and invasion assays

Motility and invasiveness were assayed using modified Boyden chambers, with 8 *μ*m pore size, polycarbonate PVP-free Nucleopore filters (Belotti *et al*, 1996; Taraboletti *et al*, 2002). NIH-3T3 supernatant (a rich source of motility stimuli) was used as the attractant and was added to the lower compartment of the chamber. For chemotaxis, filters were coated with 0.1% gelatin. For invasion, filters were coated with a thick layer of the reconstituted basement membrane Matrigel (Beckton Dickinson, Bedford, MA, USA; 0.5 mg ml^−1^) that cells have to degrade in order to reach and migrate through the filter. In this assay, invasion is prevented by inhibitors of matrix-degrading proteases, which do not affect chemotaxis (Taraboletti *et al*, 1995), therefore proving the true functional distinction between the assays of invasion and chemotaxis. Human umbilical vein endothelial cells were detached and treated with vehicle or aplidine (0.03–10 nM) for 1 h at 37°C in DMEM–0.1% BSA. Cells were then washed in DMEM–0.1% BSA, resuspended in the same medium at the concentration of 0.75 × 10^6^ ml^−1^ and added to the upper compartment of the chamber. After 4 h (motility) or 6 h (invasion), the filters were stained with Diff-Quik (Marz-Dade, Dudingen, Switzerland) and the migrated cells in 10 high-power fields were counted. Data are expressed as the percentage of control migration (vehicle-treated cells) and as IC_50_ (drug concentration causing 50% inhibition).

### Production of matrix metalloproteinases (MMPs)

Subconfluent HUVECs were treated with vehicle or aplidine (1.25–20 nM) for 1 h, washed and incubated in serum-free DMEM for 16 h, with or without phorbol 12-myristate 13-acetate (PMA, 100 ng ml^−1^). The supernatants were then collected, the remaining cells were counted and the presence of MMP-2 and MMP-9 in the serum-free supernatants was analysed by zymography (Belotti *et al*, 1996). Samples (volume adjusted according to the cell number), in 70 mM Tris-HCl pH 6.8, 10% glycerol, 2% SDS and 0.01% bromophenol blue, were applied to SDS–polyacrylamide (8%) gels copolymerised with 1 mg ml^−1^ gelatin. After electrophoresis, gels were washed three times for 20 min with 2.5% Triton X-100 at room temperature and incubated overnight in 50 mM Tris-HCI, pH 7.5, 5 mM CaCl_2_, 150 mM NaCl and 0.02% Brij-35 at 37°C. Gels were then stained with 0.5% Coomassie blue in 25% methanol and 10% acetic acid, and destained in the same solution without Coomassie blue. Supernatant of the human melanoma cells WM983A was used as a reference standard for pro-MMP-9 and pro-MMP-2.

### Cord formation

The ability of HUVEC to form capillary-like structures on a 3D layer of basement membrane (Matrigel) was tested (Belotti *et al*, 1996; Taraboletti *et al*, 2002). Ice-cold Matrigel (10 mg ml^−1^, 60 *μ*l) was layered in a 96-well plate and incubated at 37°C for 30 min to allow polymerisation. Human umbilical vein endothelial cells were treated with vehicle or aplidine (0.03–10 nM) for 1 h before the assay and then plated (2 × 10^4^ well^−1^) onto the Matrigel layer in the culture medium. Images were taken 24 h later, when cords were formed. Cord formation was quantified by measuring the total length of formed cords (Image Pro-Plus 4.5, Media Cybernetics, Silver Spring, MD, USA).

## RESULTS

### Effects of aplidine on the vascularisation of the chick CAM

**Figure 1:**
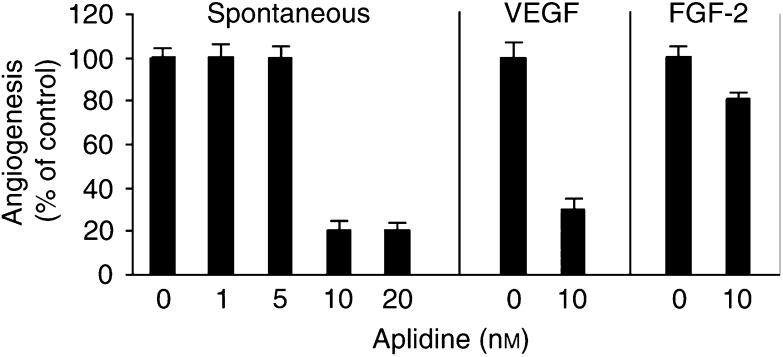
Effect of aplidine on angiogenesis in the CAM. Sponges loaded with PBS (spontaneous, basal angiogenesis), VEGF or FGF-2 and aplidine at the indicated concentration were implanted on CAMs on day 8 of incubation. On day 12, blood vessels entering the sponges or the implants within the focal plane of the CAM were counted by two observers in a double-blind manner at × 50 magnification. Results are expressed as the percentage of control response (mean and s.d., *n*=10). Vascular endothelial growth factor and FGF-2 induced a 4.7- and 4.2-fold increase in angiogenic response (38±7 and 34±4 vessels, respectively) compared to control (8±2 vessels).

The antiangiogenic activity of aplidine was investigated in the chick CAM assay, *in vivo*, a useful model to investigate the effect of compounds on both basal angiogenesis and angiogenesis induced by an exogenous stimulus (Ribatti *et al*, 2001). Aplidine, added to the CAM, affected the basal growth of vessels, inhibiting the number of blood vessels in 80% of the treated embryos (Figure 1

).

**Figure 2:**
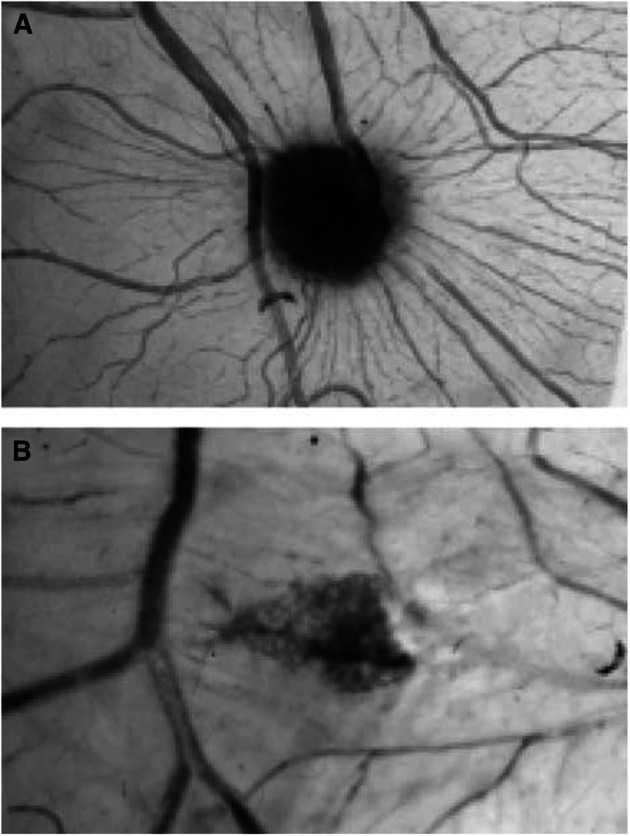
CAM of a 12-day old chick embryo incubated for 4 days with a gelatin sponge loaded with VEGF alone (**A**) or with 10 nM aplidine (**B**). Note in (**A**) numerous blood vessels converging towards the sponge, whereas in (**B**) there are very few vessels around the sponge or converging toward it. Original magnification, × 50.

Aplidine also affected angiogenesis induced by exogenous angiogenic factors added to the CAMs. Vascular endothelial growth factor or FGF-2 induced the growth of numerous allantoic vessels converging like spokes toward the sponge (Figure 2A

). Aplidine (10 nM) significantly reduced the angiogenic response induced by VEGF and, at a lower extent, by FGF-2 (Figures 1 and 2B).

**Figure 3:**
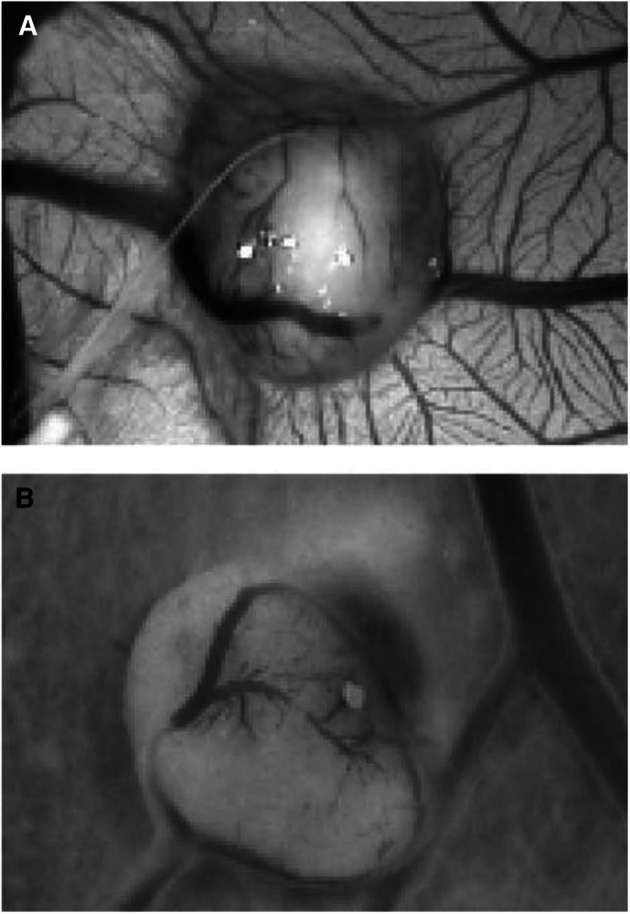
Macroscopic observations on day 12 of the effect of aplidine on tumour xenograft-induced CAM vascularisation. Fragments of the same bioptic specimen from 1A9-VS4 tumours were grafted onto the CAM on day 8 in the absence (**A**) or presence of aplidine (**B**), and incubated for 4 days. A reduction in the number of blood vessels invading the graft is observed in aplidine-treated biopsy (**B**) compared to control (**A**). Original magnification, × 50.

We next investigated whether aplidine also inhibited tumour angiogenesis induced in the CAM by tumours. The human ovarian carcinoma 1A9 and its VEGF overexpressing clone 1A9-VS4 were injected subcutaneously in nude mice. Grown tumours were collected, and fresh fragments were grafted onto the CAM, on day 8. Tumour specimens induced a strong angiogenic response, higher in 1A9-VS4 than in 1A9 grafts (number of vessels on day 12 was 30±5 and 18±4, respectively, *n*=10) (Figure 3A

). The addition of aplidine caused a significant (*P*⩽0.001) reduction in the angiogenic response induced by the tumour specimens (number of vessels was 20±4 and 11±3 for 1A9-VS4 and 1A9, respectively, Figure 3B).

These findings indicate that aplidine prevented angiogenesis *in vivo*, affecting both physiologic, spontaneous angiogenesis of the embryo, angiogenesis induced by exogenous stimuli (VEGF and FGF-2) and tumour angiogenesis. Interestingly, the normal structure of the other components of the CAM, such as the chorionic and allantoic epithelia and the fibroblasts, was not affected, suggesting a selective effect of aplidine on the vascular structures.

### *In vitro* effects of aplidine on endothelial cell functions

The *in vivo* results pointed to a direct inhibitory effect of aplidine on endothelial cells. We therefore investigated, *in vitro*, whether aplidine could affect endothelial cell functions relevant to angiogenesis. In particular, the process of angiogenesis requires endothelial cell proliferation, motility, extracellular matrix degradation and invasion through the underlying basement membrane and interstitial matrix and, finally, spatial organisation to form a network of new vessels.

**Figure 4:**
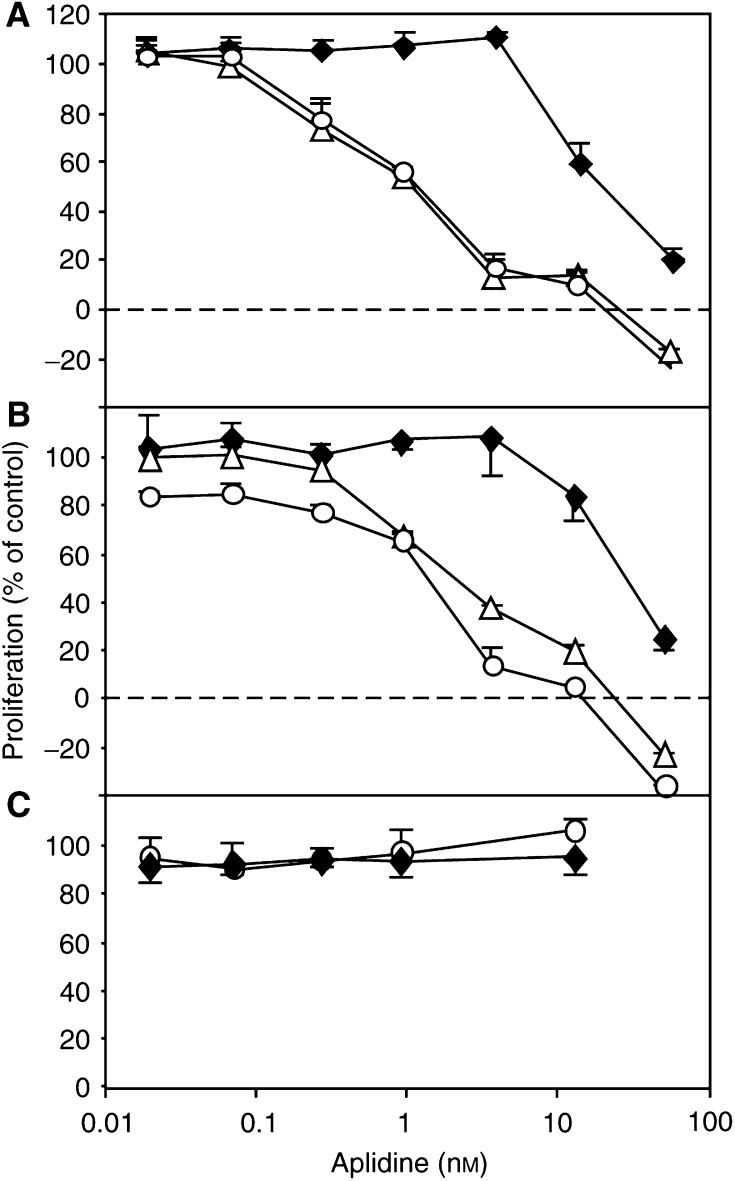
Effect of aplidine on endothelial cell proliferative response to 10 ng ml^−1^ FGF-2 (**A**) or VEGF (**B**), or on the proliferation of human proximal tubular epithelial cells HK-2 (**C**). Cells were exposed to the indicated concentration of the compound for 1 (diamonds), 24 (triangles) or 72 h (circles). Data, mean and s.d. of triplicate, are the percentage of control proliferation (vehicle alone).

Aplidine inhibited the proliferation of endothelial cells (Figure 4

). The antiproliferative effect of aplidine was observed when proliferation was stimulated by both VEGF and FGF-2. Some inhibitory effect was observed after 1 h exposure to the compound, with IC_50_ of 8.1 and 5.7 nM for VEGF and FGF-2, respectively. Exposure to aplidine for longer times (24–72 h) increased the cytotoxic effect of the compound (Figure 4). Confluent, quiescent endothelial cells were much less sensitive to the cytotoxic effect of aplidine than actively proliferating cells (IC_50_ was more than 10 times higher), even at long exposure times (72 h, not shown). In contrast, the same concentrations of aplidine that prevented endothelial cell proliferation were not active on the proliferation of the human proximal tubular epithelial cells HK-2 (Figure 4C).

**Figure 5:**
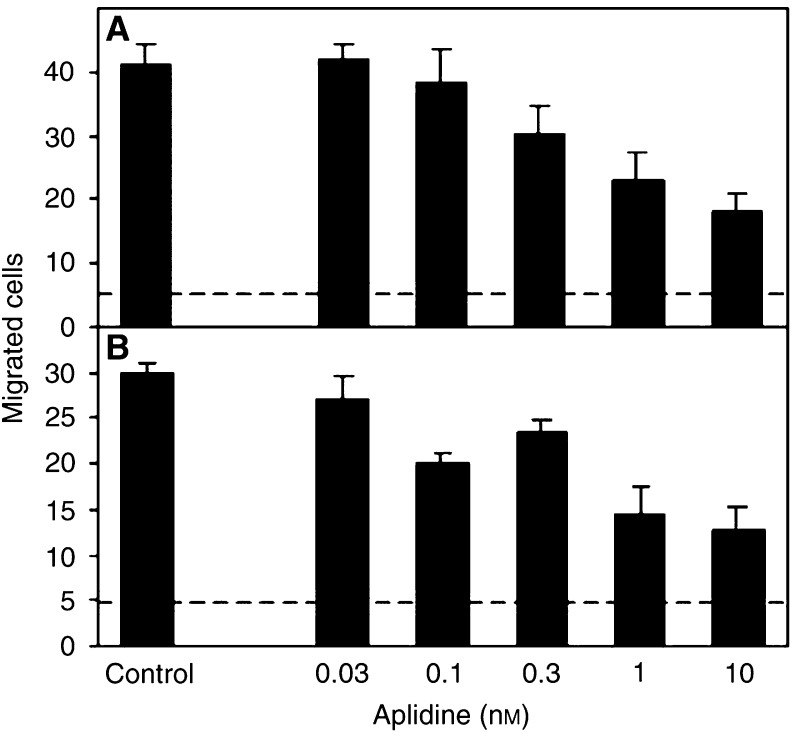
Effect of aplidine on endothelial cell motility and invasiveness. Human umbilical vein endothelial cells were treated with the indicated concentration of aplidine for 1 h and washed before the assay. Motility (**A**) and invasion (**B**) were assessed in the Boyden chamber, using supernatant of NIH-3T3 cells as the attractant. Data are the number of migrated cells in 10 high-power fields (dotted line=baseline motility).

Aplidine also affected endothelial cell motility and invasiveness, assayed in the Boyden chamber, using supernatant of NIH-3T3 cells as the attractant. Pretreatment with aplidine for 1 h resulted in a dose-dependent inhibition of cell migration, with an IC_50_ of 5.5 nM for chemotaxis and 0.9 nM for invasiveness (Figure 5

). The same results were obtained when aplidine was added to the lower compartment of the chemotaxis chamber (not shown). Aplidine also inhibited the motility and invasive response of endothelial cells to VEGF and FGF-2 (for chemotaxis, IC_50_ was 9.5 and 11.6 nM; for chemoinvasion, IC_50_ was 1.8 and 0.5 nM for VEGF and FGF-2, respectively).

**Figure 6:**
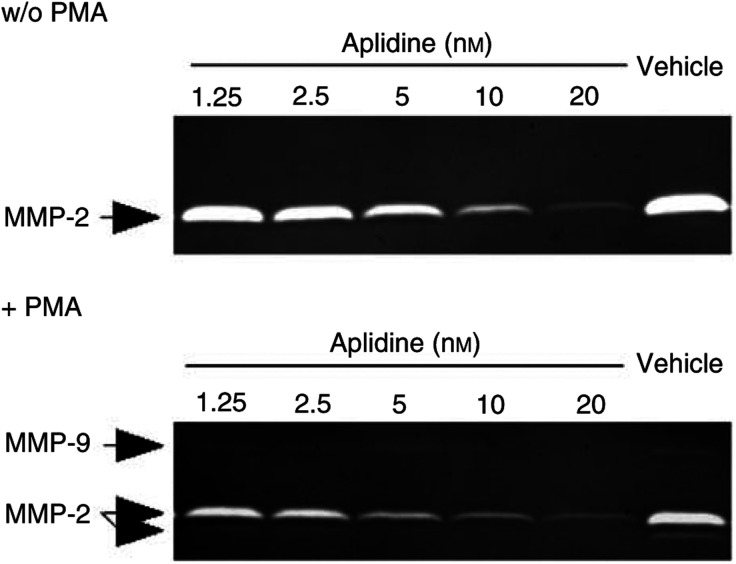
Effect of aplidine on the production of MMP-2 and MMP-9 by endothelial cells. Human umbilical vein endothelial cells were treated with vehicle or with the indicated concentration of aplidine for 1 h, washed and incubated for 16 h in serum-free medium containing or not containing PMA (100 ng ml^−1^). The supernatant was then analysed by zymography. Gelatinolytic bands corresponding to pro-MMP-9, pro- and activated MMP-2 are indicated.

Since the process of invasion requires enzymatic digestion of the matrix, we investigated whether aplidine affected the production of matrix-degrading metalloproteinases by endothelial cells. Human umbilical vein endothelial cells were exposed to aplidine (2.5–10 nM) for 1 h, washed and the serum-free conditioned medium, collected 20 h later, was tested for the presence of MMP-2 and MMP-9 by zymography. Aplidine dose dependently prevented the production of MMP-2 in nonstimulated HUVECs (Figure 6

). Treatment of HUVECs with PMA stimulated the production of MMP-9 and the activation of MMP-2. In aplidine-treated cells, even in the presence of PMA, pro-MMP-9 and activated MMP-2 were undetectable, and pro-MMP-2 was strongly reduced compared to vehicle-treated cells (Figure 6). Since we have previously reported that aplidine was able to affect the secretion of VEGF (Broggini *et al*, 2003), we also checked whether MMP secretion was affected by the compound. For this purpose, we analysed the levels of intracellular and secreted MMP-2 at different times (1, 3, 6, and 16 h) after aplidine treatment. Aplidine decreased total MMP levels, but did not change the rate of MMP secretion (data not shown), suggesting that reduction in MMP production by aplidine does not occur through perturbation of the MMP secretion process.

**Figure 7:**
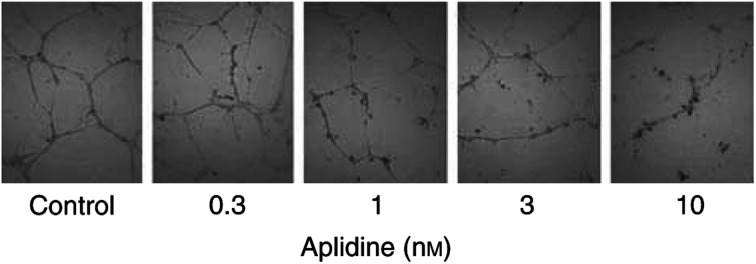
Effect of aplidine on the formation of capillary-like structures by endothelial cells. Human umbilical vein endothelial cells were treated or not with the indicated concentration of aplidine for 1 h, washed and plated onto a thick layer of Matrigel (10 mg ml^−1^) where untreated cells rapidly align, forming a network of cords. Images were taken 24 h after plating (× 40).

We finally evaluated the effect of aplidine on the alignment of endothelial cells in capillary-like structures. Endothelial cells were plated onto a thick layer of Matrigel where they rapidly align forming cords. The presence of aplidine in the assay, at concentrations ⩾1 nM, caused a concentration-dependent inhibition of cord formation (Figure 7

). At the concentration of 10 nM aplidine caused 69% reduction in the total length of formed cords.

## DISCUSSION

This study shows that aplidine, a second-generation didemnin, exerts an antiangiogenic effect *in vivo* in the CAM model and affects endothelial cell functions relevant for the angiogenic process *in vitro*. The antiangiogenic effect of aplidine was observed at concentrations achievable in plasma following the administration of the drug to patients (Armand *et al*, 2002).

The hypothesis of an antiangiogenic activity of aplidine derived from our previous finding showed that the compound inhibited the production of VEGF by tumour cells (Broggini *et al*, 2003), therefore indicating a possible effect of aplidine in reducing tumour angiogenic potential. The present study confirms that aplidine does exert a potent antiangiogenic activity, but it shows that in our models this activity is due to a direct effect on endothelial cells rather than interference with the production of angiogenic factors. Different evidences support this conclusion. (1) In the CAM assay, aplidine not only inhibited physiological angiogenesis but also angiogenesis induced by exogenous FGF-2 and VEGF, suggesting that aplidine is a general inhibitor of angiogenesis, even when the direct addition of exogenous stimuli rule out a possible effect on angiogenic factor production. (2) *In vitro* aplidine did not affect the production of VEGF by 1A9 and 1A9-VS4 tumour cells (data not shown), indicating that the antiangiogenic effect observed *in vivo* in the model of tumour grafts was not due to a reduced production of VEGF by the tumour cells. (3) Aplidine directly affected endothelial cell functions relevant to angiogenesis *in vitro*. Altogether, these findings indicate that aplidine is a general inhibitor of angiogenesis that directly acts on endothelial cells.

Our finding that aplidine affected VEGF secretion in the leukaemic MOLT-4 cells, but not in 1A9 ovarian carcinoma cells, is in keeping with the recent evidence that aplidine is able to block the secretion of VEGF in some tumour cell lines but not in others (Erba *et al*, 2003), suggesting that the compound could exert its cytotoxic activity through different mechanisms in different tumour types.

Angiogenesis is a multistep process, in which quiescent endothelial cells are stimulated by angiogenic factors to proliferate, migrate, invade the underlying matrix, form capillary-like tubular structures and, finally, organise a network of mature, functional blood vessels. Our findings that aplidine blocked endothelial cell proliferation, migration, invasion and cord formation *in vitro* suggest that the compound acts at multiple levels of the angiogenic cascade, and might explain its potent activity *in vivo*.

The molecular mechanisms of the antiangiogenic activity of aplidine still need to be defined. Different activities of aplidine have been described that might potentially contribute to its antiangiogenic effect, including effects on cell cycle/proliferation/apoptosis, as well as on protein synthesis and secretion. Aplidine inhibits endothelial cell proliferation, with an IC_50_ similar to that observed for tumour cells. Other studies reported that concentrations of aplidine that completely inhibited colony formation by cancer cells were almost ineffective on bone marrow and cord blood haematopoietic progenitor cells, nonstimulated lymphocytes and hepatocytes (Sakai *et al*, 1996; Gomez *et al*, 2001; Albella *et al*, 2002; Erba *et al*, 2003; Gajate *et al*, 2003). In agreement, we found that the concentrations of aplidine that affected endothelial cell proliferation had no relevant antiproliferative effect on epithelial cells. Therefore, at least some normal cell types are less sensitive than cancer cells to the antiproliferative and proapoptotic effects of aplidine. Our finding that endothelial cells are very sensitive to the compound therefore suggests that proliferating, angiogenic endothelial cell might be affected by aplidine in conditions in which other normal cell types are spared. This is further supported by the observation that aplidine did not interfere with the normal structure of the other components of the CAM, such as chorionic and allantoic epithelia and the fibroblasts. Moreover, our finding that aplidine is active on VEGF- and FGF-stimulated endothelial cells suggests that the compound might indeed affect the endothelium of tumour vessels, exposed to an environment rich in angiogenic and proliferating stimuli.

Several cytotoxic antineoplastic drugs are known to affect endothelial cell functions and to affect angiogenesis (Belotti *et al*, 1996; Hanahan *et al*, 2000; Klement *et al*, 2000; Kerbel and Folkman, 2002; Taraboletti *et al*, 2002). However, some of them cannot be considered true antiangiogenic compounds since the concentrations required to affect endothelial cells exceed those effective on cancer cells (Schirner, 2000; Miller *et al*, 2001). In this respect, our findings suggest that endothelial cells and cancer cells might be concomitantly affected by the same concentrations of aplidine, suggesting that the antiangiogenic effect might indeed occur in tumours and contribute to the final antineoplastic activity.

Didemnins also inhibit protein synthesis, by binding the translation elongation factor EF1-alpha (Crews *et al*, 1994). Moreover, aplidine prevents the secretion of VEGF (Broggini *et al*, 2003). It was therefore possible that these activities on protein synthesis and secretion could contribute to the antiangiogenic effect of the compound. Indeed we found that aplidine reduced the production, but not the secretion, of MMP-2 and MMP-9, two key enzymes in angiogenesis, supporting the hypothesis that inhibition of protein synthesis, although not secretion, might contribute to this activity. The effect of aplidine on MMPs might explain its higher potency (lower IC_50_) observed on invasiveness than on motility.

Aplidine, as other didemnins, has immunosuppressive activity (Sakai *et al*, 1996; Vera and Joullie, 2002). The relationship between the immune system and angiogenesis, particularly in the malignant disease, is well documented, and several immunomodulatory agents are known to affect angiogenesis (D'Amato *et al*, 1994; Turk *et al*, 1998; O'Byrne *et al*, 2000; Guba *et al*, 2002; Raje and Anderson, 2002). The relevance of this aspect in determining the antiangiogenic activity of aplidine is not clear. However, it is worth noting the striking similarity among aplidine and the apparently unrelated immunosuppressive agent rapamycin (Guba *et al*, 2002). Both compounds affect angiogenesis through a double mechanism of action: reduction in VEGF production by tumour cells and concomitant direct inhibitory effect on endothelial cell functions (Guba *et al*, 2002). Further investigations are needed to investigate whether this functional similarity implies a common molecular mechanism of action of the two agents.

In conclusion, this study indicates that aplidine has antiangiogenic activity in *in vivo* and *in vitro* models, at clinically relevant concentrations. Besides the reported activity on VEGF production (Broggini *et al*, 2003), the present study indicates an additional mechanism of action, through direct effect on endothelial cell functional response to angiogenic stimuli. Further studies are needed to further define the relative effect of aplidine on the two compartments (tumour and endothelial cells) and to identify optimal schedule and doses, to better exploit its multifaceted antineoplastic potential.
